# Smartphone gaming and frequent use pattern associated with smartphone addiction

**DOI:** 10.1097/MD.0000000000004068

**Published:** 2016-07-18

**Authors:** Chun-Hao Liu, Sheng-Hsuan Lin, Yuan-Chien Pan, Yu-Hsuan Lin

**Affiliations:** a Department of Psychiatry, Chang Gung Memorial Hospital at Linkou; b Chang Gung University, Tauyuan, Taiwan; c Department of Department of Biostatistics, Columbia University USA; d Department of Psychology, National Taiwan University; e Department of Psychiatry, National Taiwan University Hospital, Taipei, Taiwan.

**Keywords:** internet addiction, smartphone addiction, smartphone gaming

## Abstract

The aim of this study was to investigate the risk factors of smartphone addiction in high school students.

A total of 880 adolescents were recruited from a vocational high school in Taiwan in January 2014 to complete a set of questionnaires, including the 10-item Smartphone Addiction Inventory, Chen Internet Addiction Scale, and a survey of content and patterns of personal smartphone use. Of those recruited, 689 students (646 male) aged 14 to 21 and who owned a smartphone completed the questionnaire. Multiple linear regression models were used to determine the variables associated with smartphone addiction.

Smartphone gaming and frequent smartphone use were associated with smartphone addiction. Furthermore, both the smartphone gaming-predominant and gaming with multiple-applications groups showed a similar association with smartphone addiction. Gender, duration of owning a smartphone, and substance use were not associated with smartphone addiction.

Our findings suggest that smartphone use patterns should be part of specific measures to prevent and intervene in cases of excessive smartphone use.

## 1 Introduction

The increased use of smartphones in daily life has made smartphone addiction a significant social issue. Smartphone addiction and Internet addiction have both been considered technological addictions, which are defined as behavioral addictions of a nonchemical nature that involve human–machine interaction.^[1]^ Our previous study determined the 4 features of smartphone addiction: compulsion, functional impairment, tolerance, and withdrawal,^[2]^ these are also the characteristics of Internet addiction.^[3]^ The association between Internet addiction and smartphone addiction has not yet been clarified.

In 2015, an official survey by the Institute for Information Industry in Taiwan showed a 58.7% (12.25 million people) smartphone usage rate among the Taiwanese population aged 12 or above.^[4]^ One of the major features of smartphones is the ability to use multiple Internet-based mobile applications (“apps”) such as online games, social networks, and messengers. The portability of smartphones allows for shorter use periods compared with the relatively long periods of computer-based Internet use. Previous studies also suggest that smartphone addiction is associated with excessive frequency, rather than duration, of smartphone use.^[7]^ The YCPan: short use periods of smartphones results in distractions, which can lead to functional impairment, such as vehicle crashes or near-crashes.^[5]^ Epidemiological survey showed that the “screen time,” as well as the Internet usage, can affect sleep.^[6]^

Internet gaming disorder is listed as a subcategory of “substance related and addictive disorders” in Diagnostic and Statistical Manual of Mental Disorders, 5th edition (DSM-5). Gaming has received more attention than other YCPan: online activity because online gaming, particularly massively multiplayer online role-playing games, is more addictive for Internet users, leading to severe functional impairment. Smartphone gaming has increased significantly in recent years. According to 2015 gaming revenues, computers were the leading video gaming platform, with mobile devices a close second. However, no literature has focused on smartphone gaming addiction.

Nonpharmacologic responses, such as pleasure and excitement, caused by smartphones or online computer games suppressed the perception of pain in pediatric patients^[8]^ and patients with burn injuries.^[9]^ Such nonpharmacologic effects can lead to compulsive addictive behaviors and functional impairment. Unlike computer-based gaming, smartphone gaming is usually combined with multiple apps, such as social networks or messengers. The interaction between games and other apps could promote addictive behavior.

To our knowledge, no prior studies have investigated smartphone addiction or gaming in the adolescent population. The aim of this study is to explore the role of smartphone gaming and frequent smartphone use in smartphone and Internet addiction.

## 2 Methods

### 2.1 Participants and procedures

A total of 880 high school students (including those in the continuing education department) were recruited from a vocational school in central Taiwan in January 2014. Of these students, 824 were male and 56 were female. The recruitment strategy was based on the potential higher popularity of smartphone use among these students. The study was approved by the Institutional Review Board of the National Taiwan University Hospital. All participants signed an informed consent form before the study, and all clinical investigations were conducted according to the principles of the Declaration of Helsinki. Demographic data, scales of Internet and smartphone addiction, sleep quality, and characteristics of smartphone use were all measured via questionnaire.

### 2.2 Measurements

#### 2.2.1 Chen Internet Addiction Scale (CIAS)

The CIAS is a 4-point, 26-item self-reported scale that assesses Internet addiction.^[10]^ CIAS scores range from 26 to 104. Higher CIAS scores indicate a more severe Internet addiction. The internal reliability of the scale and the subscales in the original study ranged from 0.79 to 0.93, and correlation analyses yielded a positive correlation between the total scale and subscale scores of CIAS and hours spent weekly on the Internet. The diagnostic cutoff point (63/64) of CIAS for adolescents has good sensitivity and specificity,^[11]^ and we used this cutoff point to define Internet addiction in our study. We defined “Internet gaming group” if the participants choose “gaming” as their major activity on the Internet.

#### 2.2.2 The Standard and Short-Form Smartphone Addiction Inventory (SPAI and SPAI-SF)

The SPAI is a 4-point, 26-item self-report inventory, which was modified based on the CIAS^[10]^ to assess smartphone addiction.^[2]^ The SPAI demonstrated a high internal consistency (Cronbach α = 0.94) and 2-week test–retest reliability for all 4 subscales (range 0.80–0.91). The exploratory factor analysis showed that smartphone addiction consisted of 4 factors: compulsion, functional impairment, tolerance, and withdrawal.^[2]^

The SPAI was reduced into a 10-item SPAI-SF, which maintained the 4-factor structure. The total SPAI-SF score ranged from 10 to 40. The diagnostic cutoff point (24/25) for SPAI-SF was developed for college students and has acceptable sensitivity and specificity.^[12]^

#### 2.2.3 Elements of smartphone use

We defined 3 groups according to the nature of their smartphone use: the gaming predominant group, whose members indicated that the majority of their smartphone use involves gaming; the multiple-app group, whose members indicated that they used their smartphone for gaming as well as other types of apps, with no predominant use; and the reference group, whose members indicated that they use their smartphones for other functions, such as social networking, surfing the Internet, and making phone calls, but not smartphone gaming.

#### 2.2.4 Frequent use patterns and the time spent on smartphone use

To assess the total duration of the participants’ smartphone use, all participants were asked to report the average time they used their smartphone during one week and if there was a difference in the average time they used a smartphone between weekdays and weekends. If participants thought that their use pattern was too frequent to assess the total duration, they were coded as “frequent usage, very hard to estimate.” Frequent users who could not estimate the duration of their smartphone usage comprised a large proportion of our study participants. Pilot studies also demonstrated that smartphone addiction was more highly associated with frequency than duration.^[7,13]^ We therefore included the frequency of smartphone use in our model instead of the duration of its use.

#### 2.2.5 Statistical analyses

The demographics of the participants with and without smartphone addiction were compared using independent *t* test and Chi-square tests. We created a multiple linear regression model to identify the risk factors for smartphone addiction. In this model, the dependent variable was the SPAI-SF score, while the predictors included gender, the duration of smartphone ownership, smartphone gaming-predominant and gaming with multiple-apps, frequency of smartphone use, and a history of substance use. All linear regression models were created using R 3.0 (The R Foundation for Statistical Computing, Vienna, Austria) and the remaining statistical analyses were done under SPSS 18.0 for Windows (SPSS Inc. Chicago IL). *P* < 0.05 was considered statistically significant.

## 3 Results

### 3.1 Demographics

A total of 689 vocational school students (646 male) completed the questionnaire (78.3% response rate). The average age was 18.2 ± 3.6. Of the participants, 623 had a smartphone, and 155 (24.9%) were put in the “smartphone addiction group” based on SPAI-SF scores ≥25. All 4 factors of the SPAI-SF were significantly higher in the smartphone addiction group (Table 1). The smartphone addiction group spent more time on their smartphones, gaming alone and gaming with multiple apps, than the nonsmartphone addiction group (24.8% vs 18.8% and 57.5% vs 42%, respectively). There were more frequent smartphone users in the smartphone addiction group (79.1%) than in the nonsmartphone addiction group (62%). There was no significant difference in gender, time spent on the smartphone, duration of smartphone ownership, or history of substance use between the groups.

**Table 1:**
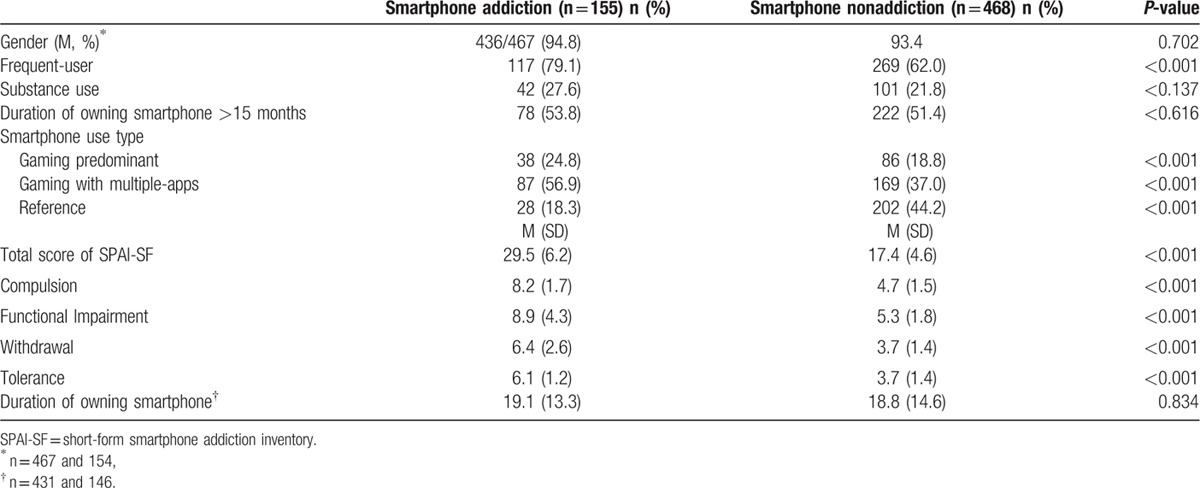
The demographic characteristics by the smartphone addiction group and nonaddiction group.

### 3.2 The role of online gaming in smartphone and Internet addictions

In the Internet addiction group (CIAS ≥ 64), 55% of participants had smartphone addiction. Only 15% of the non-Internet addiction group had smartphone addiction. The prevalent relative risk (PRR) was 3.67 (Table 2). Participants in the smartphone gaming group had a higher prevalence (32.4%) of smartphone addiction than the control group (13.2%). Similarly, there were more participants with Internet addiction in the Internet-gaming group (30.6%) compared with the nongaming group (17.4%). These results indicate that smartphone gaming (PRR = 2.75) (Table 3) is more addictive than Internet gaming (PRR = 1.82) (Table 4).

**Table 2:**
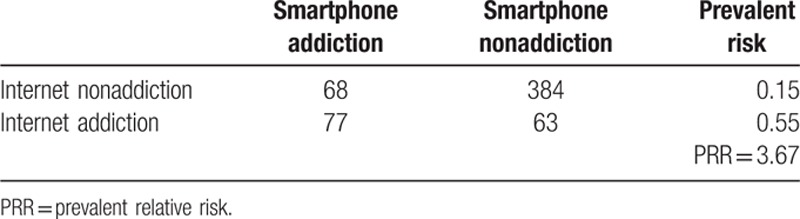
Internet addiction and smartphone addiction.

**Table 3:**
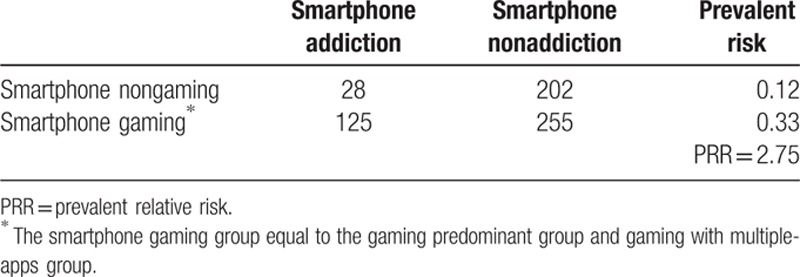
Smartphone gaming and smartphone addiction.

**Table 4:**
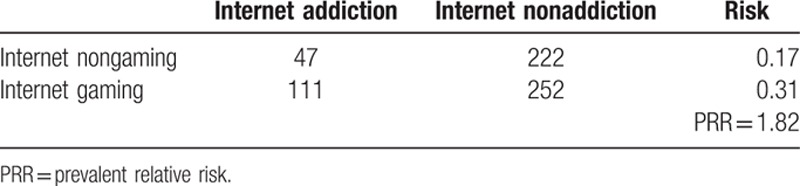
Online gaming and Internet addiction.

### 3.3 The prediction model for smartphone addiction

We fitted a multiple linear regression model with potential determinants to predict the severity of smartphone addiction (Table 5). After adjusting for other factors, the average SPAI-SF score among frequent-users was 6.87 (SD = 1.54) higher than that among the non-frequent users, indicating that frequent smartphone use played a substantial role in smartphone addiction. In addition, the SPAI-SF score among those who used gaming with multiple-apps was 5.31 (SD = 2.06), higher than for those who did not use multiple apps. The SPAI-SF score among those who played games predominantly on their phone was 4.51 (SD = 1.65) higher than for those who did not play games. There was no significant association between SPAI-SF scores and gender, duration of phone ownership, or comorbid substance use.

**Table 5:**
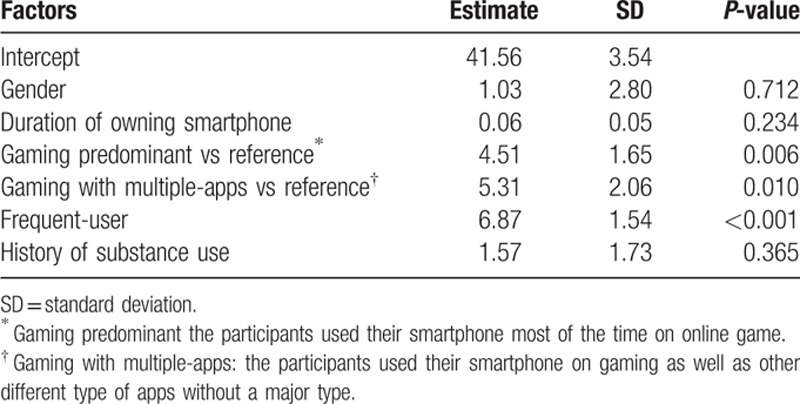
Multiple linear regression analysis using stepwise model selection to identify the correlates of smartphone addiction.

## 4 Discussion

This is the first study that demonstrates an association between frequent smartphone use patterns, smartphone gaming, and smartphone addiction in a school-based survey with a large adolescent sample size. Smartphone addicts presented with a more frequent, short-duration use pattern than nonaddicts.^[7]^ This pattern is quite different from the Internet addicts and alcoholics, who spend a great deal of time on online gaming and drinking, respectively. As a mobile Internet device, smartphone use entails different behavioral patterns compared with those of other addictive behaviors. One of the most important differences between excessive smartphone use and Internet addiction is the accessibility of smartphones, which leads to the unique behavior pattern seen with smartphone. As the craving to use a smartphone can be easily satisfied, frequent smartphone use could be an indicator of addiction.

In the highly debatable field of behavior addiction, our findings suggested the frequent use pattern and gaming associated functional impairment in smartphone addiction. The great portability of smartphone results in the unique functional impairment characterized by distraction from the frequent, short-duration smartphone use. Additionally, Internet gaming disorder has also been listed in the research criteria of the current version of DSM-5. The work group focused on gaming because it was the most well studied and arguably problematic form of Internet use at the time.^[14]^ Even though many apps involved in smartphone using, it is still critical to identify the role of gaming in smartphone addiction.

This study revealed that smartphone gaming plays an important role in smartphone addiction. The availability of Internet gaming on computers was limited by the quality of the device and Internet access. That is not the case in smartphone gaming. Smartphone gaming posed a higher risk for smartphone addiction than Internet addiction. Our findings showed that smartphone gaming, with and without the use of multiple apps, increased the risk of smartphone addiction. Smartphone addiction criteria are more similar to those for a generalized Internet addiction than Internet gaming disorder, as described in the DSM-5.^[7]^ Smartphone gaming allows players to connect with other players through the Internet using social networking apps. These apps promote the game to the publics and enhance addictive behaviors. Prior evidence has indicated that smartphone addiction is a multiple-app addiction, which is similar to the case of massively multiplayer online role-playing games. In conclusion, general gaming is the primary addictive behavior, but the nature of multiple-app use makes the symptoms of smartphone gaming addiction different from those of Internet gaming disorder.

Substance use was unrelated to smartphone addiction in our study. This finding was consistent with a Swiss study of adolescents, which did not find an association between excessive smartphone use and alcohol or tobacco consumption.^[15]^ However, Internet addiction was significantly associated with problematic alcohol use in adolescents^[16]^ and young adults.^[17]^ The shared personality characteristics seen in individuals with problematic Internet use and alcohol dependence^[17,18]^ may not play a role in smartphone addiction. In our study, the duration of owning a smartphone was also unrelated to smartphone addiction. However, the average length of ownership in our study was less than 2 years. The association between smartphone exposure time and smartphone addiction should be investigated by a longitudinal study.

Several methodological limitations should be noted when interpreting our findings. First, the participants consisted of mostly male vocational school students. A previous survey revealed smartphone addiction was positively related to female gender, which may related to the different favorite Internet-based activities.^[19]^ However, the male-predominant population in our study restricted the possibilities for generalizing our findings. Second, the cross-sectional design of our study limited the possibility of making causal inferences about the relationship between smartphone addiction and smartphone gaming or frequent use patterns. Third, smartphone addiction was identified with self-reported questionnaires, rather than a diagnostic interview conducted by professionals. Apps that were developed to automatically record smartphone use data could be an alternative tool to confirm the frequent use patterns and correct the “time-distortion effect.”^[7]^

In conclusion, smartphone gaming and frequent use patterns were associated with smartphone addiction. These findings suggest that parents, school counselors, psychiatrists, and other mental health professionals should take steps toward preventing smartphone addiction by considering use patterns and gaming when developing early detection tools and intervention programs in school or community settings.
