# Smartphone gaming and frequent use pattern associated with smartphone addiction: Erratum

**DOI:** 10.1097/01.md.0000504796.68207.67

**Published:** 2016-10-21

In the article “Smartphone gaming and frequent use pattern associated with smartphone addiction”,^[1]^ which appeared in Volume 95, Issue 28 of *Medicine*, several typos have been corrected. The article has since been corrected online.
